# The New International Health Regulations and the Federalism Dilemma

**DOI:** 10.1371/journal.pmed.0030001

**Published:** 2005-12-20

**Authors:** Kumanan Wilson, Christopher McDougall, Ross Upshur

## Abstract

The recent revision of the International Health Regulations, say Wilson and colleagues, is both long overdue and eminently necessary to face the challenges of an increasingly globalized world.

In the aftermath of severe acute respiratory syndrome (SARS) (Figure 1) and in anticipation of avian flu, the international health community has recognized that pandemic planning and response is an inherently multigovernmental concern. The ability of pathogens to cross borders and rapidly spread around the globe requires highly coordinated public health responses that involve the cooperation of local, regional, national, and supranational governments (Figure 2). The understanding of this reality has informed the current International Health Regulations (IHR) revision process.

**Figure 1:**
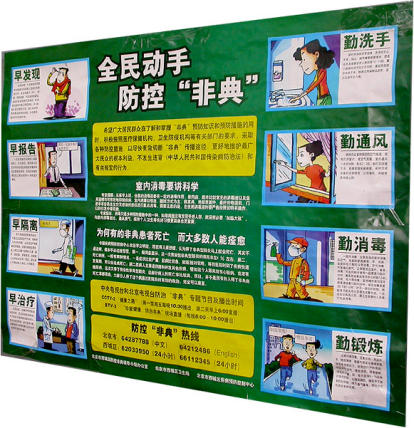
“8 Steps Towards SARS Prevention”—Public Information Poster Issued by the Chinese Government, 2003

**Figure 2:**
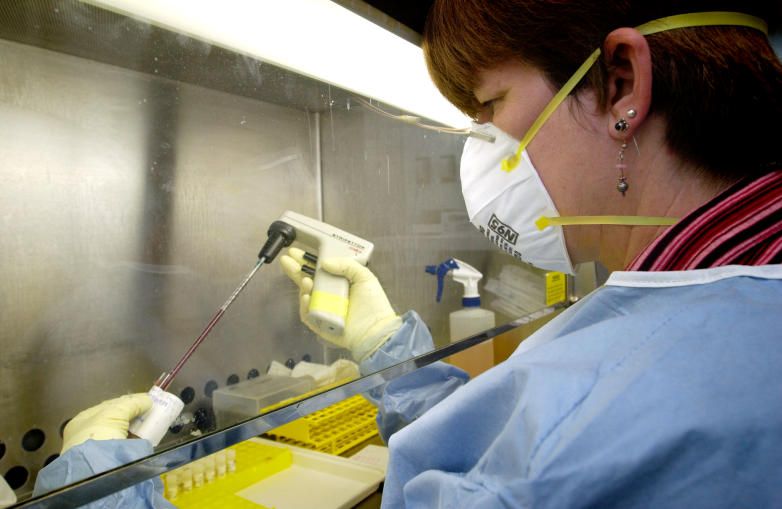
Processing SARS Specimens at the Special Pathogens Branch of the CDC The Centers for Disease Control and Prevention (CDC) worked closely with the WHO and other partners, as part of a global collaboration to address the SARS outbreak. (Photo: James Gathany/CDC Public Health Image Laboratory)

Approved in May 2005, the revised IHR have increased the disease surveillance requirements of “states parties” in an effort to better inform the pandemic response process and to protect the health of the global community [1]. Furthermore, the revisions have also outlined recommendations that the World Health Organization (WHO) could issue if an outbreak originating in one country is perceived to be a threat to other countries. The revision of the IHR is both long overdue and eminently necessary to face the challenges of an increasingly globalized world [2]. The practical implementation of these proposals, however, may encounter obstacles. This is particularly true for those WHO member nations that have federal systems of government (federations), and could ultimately threaten their ongoing support of the new regulations.

## Federalism and Public Health Response

Federalism is a type of political system in which the advantages of shared rule are combined with those of regional government [3]. Countries with federal governments make up about 40% of the world's population, and include the second most populous country (India) and the world's largest economy (United States) [4]. Federal systems of government offer many advantages, including allowing for the distinctiveness of the regions within a nation to be recognized and for region-specific policy approaches to be developed. However, one of the limitations of federations is that the division of powers can create an obstacle to the development of centralized approaches to national challenges. Such scenarios can arise when the country's constitution distributes the key powers in question to the regions. This characteristic of federal systems poses a dilemma when international treaties are signed by a federal government, but the cooperation of regional governments (states, provinces, etc.) is necessary for compliance with the treaty.

Canada's experience with SARS outlines the challenges of such constitutional division of powers when it comes to managing public health crises. During SARS, the Canadian federal government's ability to obtain data from the Province of Ontario was dependent on voluntary transfer, since the management of infectious disease outbreaks falls under provincial jurisdiction [5,6]. Reviews of the response to SARS showed that the transfer of data from the provincial government to the federal government was a key obstacle to the management of the crisis and, in particular, limited the federal government's ability to effectively communicate the status of the outbreak to the WHO (Problem 7 of [7]).

The United States encountered similar jurisdictional problems when developing strategies to address the threat of bioterrorism following the 2001 anthrax attacks, because public health is primarily within the jurisdiction of the states [8,9]. Concerns about the inadequacy of some state public health legislation at the time provided the impetus for the development of a Model State Emergency Health Powers Act [10].

In Australia, powers over emergency response to public health crises also primarily reside at the state level, with the federal government having limited authority except for quarantine. The development of a coordinated Australian approach to managing new infectious threats has thus been a challenge, and there is potential for confusion over who has authority in the event of a public health emergency that crosses state borders [11].

Adding to the challenges of developing effective intergovernmental approaches to disease outbreaks in federations is their multidimensional nature. The response to an infectious outbreak could involve issues of national security, emergency response, environmental protection, and food and water safety. Powers over these areas may be differentially allocated across the various orders of government. Such a scenario could produce conflict or confusion when attempting to determine which order of government has the ultimate authority over the management of the outbreak. This in turn may contribute to a failure to adequately manage an outbreak and to the spread of the outbreak across borders within a country, and potentially into other countries.

## The New International Health Regulations

The revisions of the IHR create important new challenges to all countries, and in particular to those with federal systems of government (Table 1) [12]. The new IHR require all states parties to designate representatives to implement the surveillance, response, and notification requirements of the regulations. These requirements cover all jurisdictions from the community level to the national level. While the previous version only applied to three infectious diseases, the new IHR apply to “all events that may constitute a public health emergency of international concern,” and a decision-making instrument to assist in the identification of such events is included in the regulations. Notifiable events within a state are to be reported by a “national IHR Focal Point” to the WHO within 24 hours. States are also responsible for strengthening their surveillance system and are required to complete both a capacity assessment within two years of the approval of the revised Regulations, and the development of public health infrastructure that ensures full compliance within five years of the entry into force (in June 2007) of the regulations.

**Table 1:**
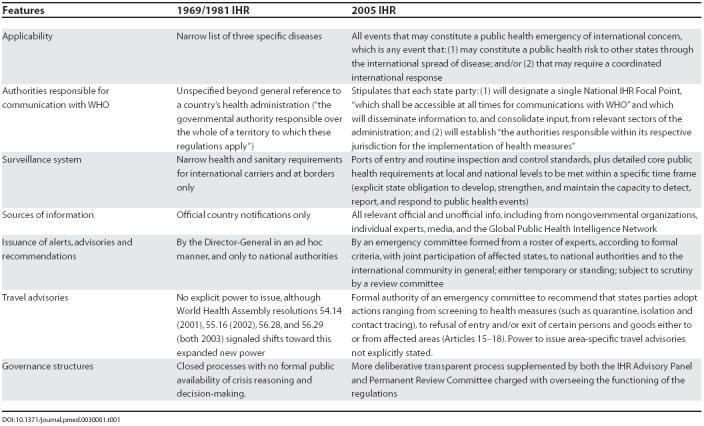
Some Changes to the IHR That Are Relevant to Federations

The new IHR also explicitly outline new WHO powers, which include an information-gathering prerogative that is not limited solely to official state notifications or consultations, but which covers all “the available scientific evidence and other relevant information”. The WHO is also empowered to share information with other states parties if an affected state “does not accept the offer of collaboration” and “when justified by the magnitude of the public health risk”. The revisions also formally empower the WHO to issue temporary and standing recommendations if an outbreak is classified as a public health emergency of international concern. These could include recommendations to issue travel restrictions for persons from affected areas. The recommendations would ideally be made with the consent of the affected country, although provisions exist for such action in the absence of the target member state's consent.

## Federalism and the New International Health Regulations

The realities of federations, and the fragmentation of powers within them, could become particularly problematic when attempting to operationalize the new IHR. This is particularly true of the surveillance and reporting requirements, and it is of particular concern given the implications of new WHO powers. There is a real concern that federations may not be able to comply with the IHR, which could result in the issuance of temporary recommendations that would penalize federations for political and administrative features that they perceive to be beyond their control.

While a primarily unitary state may have sufficient centralized powers to ensure that the surveillance and reporting requirements embodied in the new IHR are met, the allocation of powers within federations may not permit this. For example, the IHR revisions require all member nations to notify the WHO “within 24 hours of assessment of public health information, of all events which may constitute a public health emergency of international concern within its territory…as well as any health measure implemented in response to those events”. However, federal governments may not have the authority to collect the data necessary for reporting to the WHO and the transfer of data from the affected regions may not occur voluntarily. While, according to the new IHR, the federal government can designate regional representatives to carry out some of the measures, the government is ultimately dependent on cooperation from these regional authorities that may not necessarily be forthcoming.

There are several reasons why regional governments, for example, may not want to provide complete information on the nature of an outbreak. These include concern about the impact of disclosure on their economy, aversion to federal scrutiny, concerns about stigmatization, and a fundamental belief that the issue being addressed is within their jurisdiction. Failure to report could have significant consequences, most importantly the delay of national and international responses to prevent the spread of the disease. The resistance of regional governments to sharing of information could leave a country susceptible to measures introduced by the WHO, particularly since the WHO now has authority to conduct surveillance and to utilize information gathered from nongovernmental sources through the Global Public Health Intelligence Network [13,14].

The issuance of travel advisories, as occurred during SARS, or of temporary recommendations to member nations not to accept travelers from an affected region, could have damaging effects on the economy of that region. The perception in Canada was that the SARS travel advisory may not have been warranted, and had a serious negative impact on the economy of Toronto [15,16]. In developing countries where tourism is essential to the national economy, such an advisory could be catastrophic. Federations could view the use of such measures by the WHO as unnecessarily punitive and an invasion of national sovereignty. Ultimately, this could lead to a lack of support for the WHO, the IHR and other global strategies for disease control [12].

## Federalism and International Agreements

The United States, recognizing the challenges the IHR could pose for federations, had requested the insertion of a clause that would acknowledge the unique governance structures of federations. The decision not to include such a clause in the revised IHR prompted the United States to notify that they intend to submit a statement of reservation, specifically commenting that they will “implement the IHR in a manner consistent with (their) federal system of government” [17].

The challenges posed by the IHR to federations are not unique, however, as federations are often confronted with difficulties in implementing international agreements. For example, the GATT/WTO agreement included a federal clause which states that “[e]ach contracting party shall take such reasonable measures as may be available to it to ensure observance of the provisions of this Agreement by the regional and local governments and authorities within its territory”. However, there has been variability in the interpretation of this clause, with certain countries claiming that this requires the use of any constitutional power available to adhere to the agreement, and others arguing that this clause should not allow internal federal structures to be compromised [18,19]. The Kyoto Protocol to the United Nations Framework Convention on Climate Change has generated similar challenges to implementation in federal states, and it is notable that two key federations, the US and Australia, have not ratified the treaty [20].

The problems federations have with compliance with international treaties can occur at several levels. First, as already mentioned, the necessary powers to ensure compliance with a treaty may not fall within the jurisdiction of the federal level of government. While in many federations, the federal power to sign treaties may permit them to override the jurisdiction of regional governments, this is not necessarily always the case. And in scenarios in which the federal government does have the necessary constitutional authority, they may choose not to exercise it because of political concerns about creating conflict with regional governments. Even in scenarios in which the federal government has the necessary legislative power and does choose to exercise it, regional governments may not be able to cope with the financial and/or practical burdens of compliance [21]. This latter issue, of unfunded mandates, is not unique to federal states and constitutes a challenge that all WHO member nations will need to address when determining how to implement the surveillance requirements of the new IHR.

## Guidance to Federations

It is apparent that this “federalism dilemma” will need to be addressed, both by member nations and by the WHO, if the revised IHR are to be implemented successfully. It is primarily incumbent upon federations, as responsible members of the international community, to take every measure available to ensure that they can comply with the new IHR. These measures would include using what constitutional means may be available to centralize necessary public health powers concerning surveillance and outbreak response, making efforts to establish effective collaborative intergovernmental arrangements, and developing appropriate public health capacity at the local level. The intergovernmental acrimony to which federations are susceptible would clearly not be acceptable if, at a time of crisis, it produced a dysfunctional response that resulted in the international spread of disease.

The WHO, in turn, must make efforts to assist federations in this regard. These efforts could include the provision of guidelines on strategies federal governments can use to address some of the challenges we have described. For example, the constitutions of some federations may have unexploited powers that federal governments could utilize to gain the necessary authority. Constitutions are often interpreted in a flexible manner by courts, in recognition of the realities of a changing world. The threat of pandemic infections could be taken strongly into consideration when courts are interpreting the use of federal powers. Of course, there are important limitations to this strategy, including the fact that any constitutional interpretation will need to strike a balance between new public health powers and respect for fundamental human rights and traditional allocations of government power, both of which may constrain the expansion of federal authority. Furthermore, heavy-handed, top-down approaches to managing disease outbreaks are not ideal, given the critical importance of local and regional public health activities. Ideally, responses would be a collaborative venture between orders of government.

To facilitate such collaboration, federal governments could enter into agreements with regional governments to ensure cooperation on matters such as the timely and adequate transfer of data. The likelihood of adherence to such agreements would be greatly enhanced if conditional funding were provided by the federal government to assist in developing the necessary surveillance and response infrastructure at the local level. This option is particularly important to consider because adequate federal powers will have no effect in the absence of adequate surveillance infrastructure or public health capacity. Governance strategies must go hand in hand with effective surveillance and the development of necessary public health capacity [22].

## Conclusion

Given the importance of the IHR revision process, every effort should be made to ensure that member nations can comply with the new requirements. The size and power of several federations in the World Health Organization require that the particular nuances of their governance structure be acknowledged and respected. Failure to do so could threaten the long-term support of the IHR by key federations, such as the United States, India, and Russia, which would undermine their fundamental objective of protecting the global community. Ultimately, however, it is the responsibility of federations to make the appropriate adjustments in their approach to public health governance so that they can effectively identify, respond to, and communicate information on disease outbreaks.

## Abbreviations

IHR: International Health Regulations
SARS: severe acute respiratory syndrome
WHO: World Health Organization
